# Assessment of clinical outcomes and patient response to gingival depigmentation using a scalpel, ceramic bur, and diode laser 980 nm

**DOI:** 10.1007/s00784-023-05310-w

**Published:** 2023-10-25

**Authors:** Faten Fawzy Mikhail, Hala El Menoufy, Naglaa Shawki El Kilani

**Affiliations:** 1 Surgery & Oral Medicine Department, Oral & Dental Research Institute, National Research Centre, 33 El Buhouth St., Ad Doqi, Dokki, Giza, 12622 Egypt; 2 Laser Research Centre, Faculty of Dentistry, Misr University for Science and Technology (MUST), 6Th of October City, Giza, Egypt; 3 Oral Medicine, Periodontology, Diagnosis, and Radiology Department, Faculty of Dentistry, Al-Azhar University, Cairo, Egypt

**Keywords:** Scalpel, Ceramic trimmer bur, Diode, Laser, Gingival hyperpigmentation, Melanin

## Abstract

**Objective:**

This research compares the clinical outcomes of gingival depigmentation procedures with conventional scalpel, ceramic trimmer bur, and diode laser techniques.

**Materials and methods:**

Twenty-four individuals with physiologic gingival hyperpigmentation received random allocation to one of three treatment groups: scalpel, ceramic bur, or diode laser. Pain score, operation time, bleeding index, degree of epithelialization, wound healing, Dummett-Gupta Oral Pigmentation Index (DOPI), and Takashi Index score changes were all investigated at different time points.

**Results:**

At 12-h follow-up, significant variations in pain scores were seen between the laser and scalpel groups (*p* = 0.003) but not between the laser and ceramic bur groups. The diode laser group completed the procedure significantly quicker than the scalpel and ceramic bur groups (*p* = 0.004 and *p* = 0.001, respectively). The ceramic trimmer bur and diode laser groups showed significantly less bleeding tendency than the scalpel group. Wound healing and the degree of epithelialization were similar in all groups. DOPI and Takashi indices significantly decreased compared to baseline in all groups, with no significant difference recorded between all groups.

**Conclusion:**

While diode lasers are a safe and effective treatment option for gingival hyperpigmentation, providing optimal aesthetics with reduced discomfort to patients, a ceramic trimmer bur can also be used as a simple and affordable alternative to a laser in gingival depigmentation procedures.

**Clinical relevance:**

Gingival hyperpigmentation is a major aesthetic issue for many individuals. Laser and ceramic trimmer bur treatments produce equivalent aesthetic outcomes for gingival hyperpigmentation.

## Introduction

Gingival color plays a crucial part in facial aesthetics and contributes to the entire appearance of an ideal smile [1]. Various factors influence gingival color, including the tissue’s blood supply, epithelial thickness, keratinisation degree, and particles like melanin, melanoid, oxyhemoglobin, carotene, and iron [2].

Melanocytes originating from the neural crest are responsible for melanin production. Melanocytes typically reside in the epithelial basal and suprabasal layers. In humans, they establish close connections with 30 to 40 keratinocytes, enabling melanocytes to release melanin into the keratinocytes. Melanocyte activity, along with other variables, predominantly determines the level of pigmentation [3].

Certain physiological or pathological factors have the potential to trigger gingival hyperpigmentation. Although physiologic hyperpigmentation is entirely harmless, some individuals encounter aesthetic concerns consequently, with black gums being the prevailing manifestation [4]. These individuals typically aspire to eliminate gingival coloration to enhance the aesthetic appeal of their smile. Gingival hyperpigmentation (GHP), or “black gum,” constitutes one of the contributing elements that significantly impact how someone looks when they smile [5]. Several treatments, such as gingivectomy, mucosal removal with a scalpel, abrasion techniques, free gingival grafts, chemical approaches involving caustic chemicals, electrosurgery, cryotherapy, and newly developed lasers, have been used to achieve pigmentation-free gingiva [4].

The scalpel technique, which involves eliminating the gingival epithelium and a layer of connective tissue, is one of the earliest and most popular methods for gingival depigmentation. Subsequently, this will allow the stripped connective tissue to heal by secondary intention, producing an epithelium devoid of melanin pigmentation [6]. The wound heals faster with this procedure but is more painful [7–9].

Ceramic trimmer burs were initially introduced in dentistry for gingivoplasty. However, recently, they have also been used to treat gingival hyperpigmentation. These trimmers are composed of mixed ceramics, including zircon-dioxide ceramic that has been partially stabilized with yttrium and ceramics made of aluminum. They offer a smooth, delicate incision, and the heat generated ensures effective hemostasis, minimal bleeding, and almost no risk of necrosis [10].

Since the 1970s, lasers have been extensively used in medical and dental fields for various surgical techniques [11]. It has been claimed that employing different lasers is dependable, safe, and practical, often resulting in reduced postoperative pain and bleeding. Additionally, it can sculpt, cut, and remove gingival tissues [12].

Two proposed approaches have been suggested, based on the specification of wavelength absorption, for laser gingival depigmentation. In the first surgical/ablative method, the gingival epithelium, which contains melanin, is ablated. This method can be employed at any surgical wavelength. The second method, on the other hand, is non-invasive or non-ablative. It involves the degranulation or denaturation of melanin without removing the gingival epithelium. This is achieved by using a laser with a particular wavelength, like a visible diode laser (445 nm) or a near-infrared diode laser (810 nm) [13].

Several studies have compared using a ceramic trimmer bur and a diode laser for gingival depigmentation. However, neither technique yielded significantly different results regarding aesthetic satisfaction or pain, as measured by the Visual Analog Scale (VAS) [14, 15].

According to the existing literature, there needs to be a consensus on which technique is superior. Thus, the present study aims to compare the clinical outcomes of gingival depigmentation operations with a traditional scalpel, ceramic trimming bur, and diode laser techniques.

## Materials and methods

### Sample size calculation

The sample size was calculated using PS software. Regarding the primary outcome, pain at 7 days, we clarify that six patients per group would be an adequate sample size for the study, with a total sample size of 18 patients (3 groups). The power is 80%, the *α* error probability is 0.05, and the effect size is 0.82. An increase in the number of patients was carried out from 18 to 24 patients to compensate for the predicted missing data, which was 25%. The relevant variable’s mean and standard deviation (mean ± SD) were used to assess the magnitude of the effect to be found [16].

### Recruitment and eligibility criteria

Twenty-four patients older than 18 were selected for the study from the Periodontics Department, Faculty of Dentistry, Misr University for Science and Technology, in 6 October City, Egypt, 2023. The study was conducted in conformity with the Code of Ethics of the World Medical Association (Declaration of Helsinki) for experiments including humans. Each patient had to complete a written consent form for the procedure and follow-up before participation. The Misr University for Science and Technology’s Institutional Review Board (MUST-IRB) approved this study on March 29, 2023, with an ethical approval number (No. 2022/0059).

The inclusion criteria for this study consisted of systemically healthy patients who had gingival hyperpigmentation related to the aesthetic region, good oral hygiene, and a desire for aesthetic improvement. Patients who were smokers, pregnant or lactating women, experienced keloid scarring following surgery, or whose gingival hyperpigmentation was linked to another syndrome or illness were excluded.

A comprehensive medical and dental history was gathered for each patient. For all the selected patients, comprehensive scaling and root planning and oral and general examinations were performed, and they were then provided with oral hygiene advice.

The eligible patients were allocated to three equal groups (8 patients each). The depigmentation procedure was performed for the maxillary and mandibular anterior regions (from right canine to left canine) using a scalpel (group 1), a ceramic trimmer bur (group 2), and a diode laser 980 nm (group 3).

### Treatment protocol and interventions

Before the procedure, each patient received infiltration anesthesia with 1.7 mL/jaw (Ubistesin™ 1/200,000, 3 M ESPE AG, Germany)^1^ injected into the upper and lower anterior vestibular regions. The subjects received random allocation to one of three treatment groups by the coin-flip method. Each patient was given a number. Thus, they ranged from 1 to 24 patients. Subsequently, two coin flips were performed for each patient, and the outcomes were recorded. Based on the results of these coin tosses, the patients were divided into their respective treatment groups. For illustration, we followed the guidelines below:

- Group 1, if both coin flips were heads (HH).
- Group 2, if both coin flips were heads (TT).
- Group 3, if both coin flips were heads (HT or TH).

The total count of patients allocated to each category was subsequently tallied. In an imbalanced distribution, wherein one group possessed a considerably larger number of patients than the others, it became necessary to reiterate the randomization procedure until attaining a more equitable distribution.

**Fig. 1:**
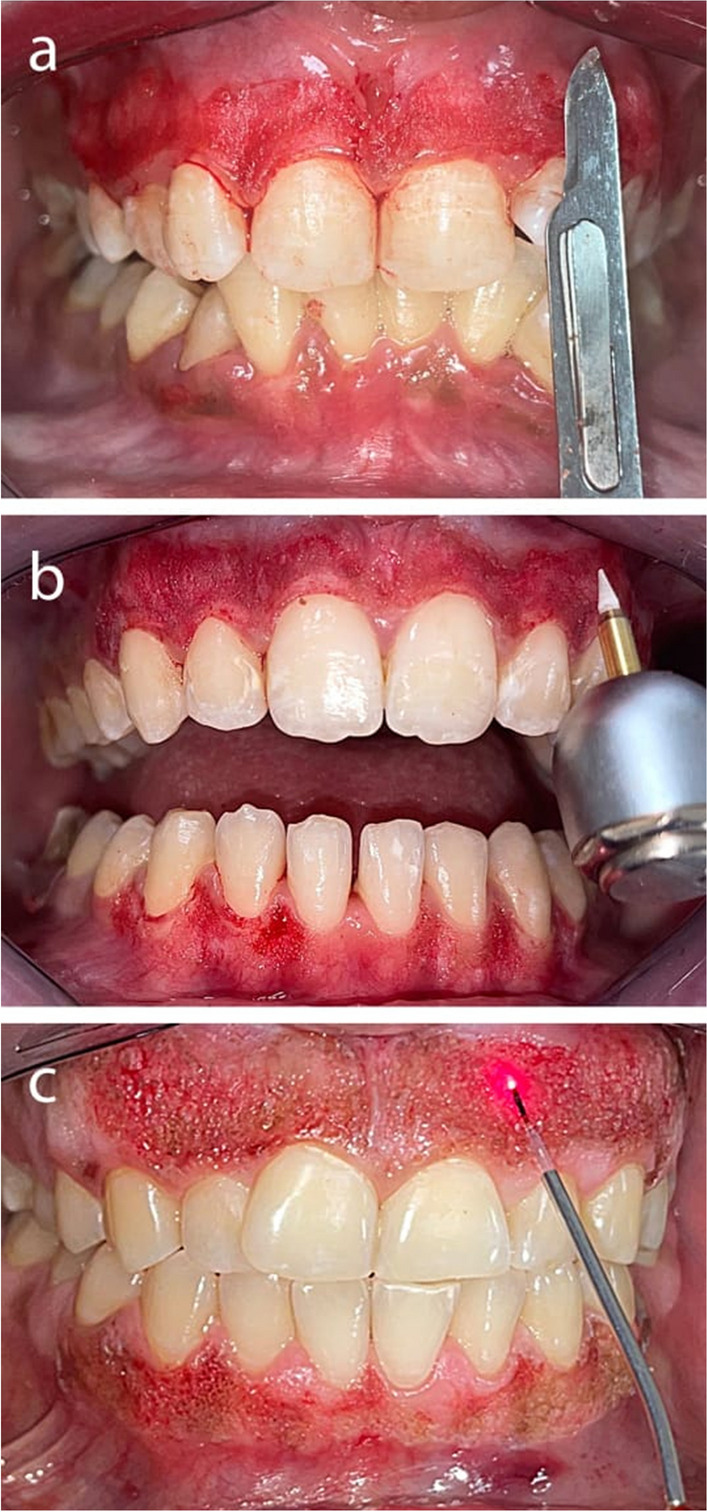
**A** Depigmentation was performed by a surgical scalpel; **b** depigmentation was performed by a trimmer bur; and **c** depigmentation was performed by a diode laser

The subjects and operators for the laser group put on eyewear in accordance with the laser’s operating wavelength. The following gingival depigmentation techniques were then applied at random to the treated areas:

- Group 1 (surgical scalpel): Using surgical scalpel blade No. 15, the whole epithelium and a layer of connective tissue were stripped off up to all of the apparent pigment was eliminated from the free gingival margin to the mucogingival junction [17] (Fig. 1a). A pressure pack was directly applied to the operative region to control bleeding.
- Group 2 (ceramic trimmer bur): A ceramic soft tissue trimmer bur (DFS Diamon Precicut® tissue trimmer, Germany)^2^ was utilized at high-speed 300,000 rpm without water coolant spray, according to the manufacturer’s guideline and previous works [15], to eliminate the whole epithelium and a layer of connective tissue. Saline irrigation was then applied to the exposed surface (Fig. 1b).
- Group 3 (diode laser): A diode laser with a wavelength of 980 nm (Doctor Smile Diode Laser, LAMPDA, Italy)^3^ was used to perform the depigmentation procedure. The depigmentation treatment was executed utilizing specific parameters: a 400-μm initiated tip (8 times, each setting, employing articulating paper), an average power of 1 W, continuous mode, a duty cycle of 100%, a power density of 796.17 w/cm^2^ at the tip of the fiber, and the absence of water or air [18]. The procedure involved the placement of the laser tip in contact with the gingival surface at an angle of approximately 30° to prevent overheating. Laser ablation was undertaken using a technique similar to brushing or painting, commencing at the mucogingival junction and progressing towards the free gingival margin, from the canine tooth to the contralateral tooth of the jaw. Whenever the interdental papilla exhibited hyperpigmentation, it was also incorporated into the ablation procedure (Fig. 1c).

To improve visibility and cool the tissue during the ablation process, the tissue fragments were wiped away using sterilized gauze dipped in a normal saline solution, with special attention paid to protecting tooth surfaces. The tissues were eliminated while keeping 1 mm away from the gingival margin and through the thickness of the keratinized attached gingiva to reduce the risk of gingival recession. Thus, adequate attention was exercised to reduce the thermal adverse effects of the laser on the surgical area and to prevent unneeded tissue damage following laser use. Ventilation with suction was done during the procedure. The laser procedure was maintained until no pigmentation was apparent upon observation.

No periodontal pack was used to ensure appropriate assessments and follow-up for pain and wound healing. The participants were instructed to refrain from eating spicy and hot foods during the first-week postoperation.

### Postoperative assessments

**Fig. 2:**
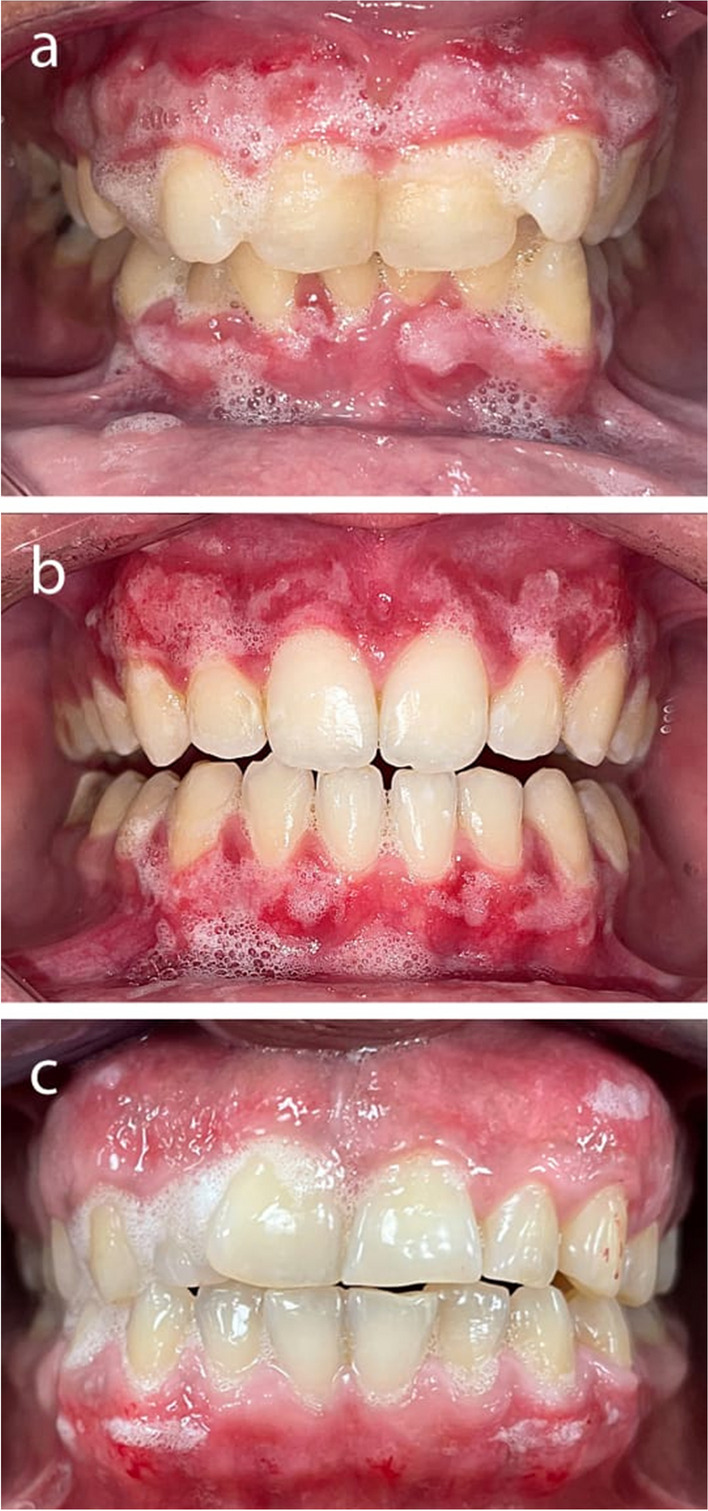
**A** Hydrogen peroxide test of the surgical scalpel group after 4 days; **b** hydrogen peroxide test of the ceramic trimmer bur group after 4 days; **c** hydrogen peroxide test of the diode laser group after 4 days

The subsequent criteria were assessed throughout and following the procedure in the study groups:

1. **Pain:** Pain was assessed using the Visual Analog Scale (VAS), depicted by a 10-cm horizontal line with one of its ends denoting no pain and the other denoting severe pain (score 10). Patients’ scores of pain were accurately requested as follows: (0) there is no pain; (1–3) experienced mild pain; (4–6) experienced moderate pain; and (7–10) experienced severe pain. The VAS was assessed thrice: 12 h postoperation and on the 4th and 7th days postoperatively.
2. **Bleeding** will be measured during the surgery. A clean, dry wound will be scored as “0 = no bleeding,” a wound with slight oozing of blood will be scored as “1 = oozing,” and a wound bleeding enough to fill the mouth with blood is frequently scored as “2 = active bleeding” [19].
3. **Duration of the treatment:** The operation time was measured in minutes, starting as soon as the procedure began and terminating when all pigment had been removed from the treated region.
4. **The degree of epithelialization** was evaluated visually on the 4th and 7th days postoperatively, where the region to be tested was dried, and 3% hydrogen peroxide (H2O2) was administered for wound healing purposes (Fig. 2) [20]. The absence of the peroxide test indicated complete epithelialization. Evaluation standards were as follows:
  Negative ( −): No bubble formation (complete epithelialization)
  Positive ( +): Bubble formation (incomplete epithelialization)

5. **Wound healing** was assessed on the 7th day postoperation based on the Healing Index of Landry, Turnbull, and Howley [21–23].
6. **Gingival pigmentation was assessed using**
  **Dummett-Gupta Oral Pigmentation Index (DOPI) **[24]
    0: No clinical pigmentation (pink tissue)
    1: Mild clinical pigmentation (light brown)
    2: Moderate clinical pigmentation (medium brown)
    3: Heavy clinical pigmentation (deep brown to blue black)
  **Takashi Index **[25]
    0: no pigmentation
    1: solitary unit (s) of pigmentation in papillary gingiva without extension between neighboring solitary units
    2: formation of a continuous ribbon extending from neighboring solitary units

**Fig. 3:**
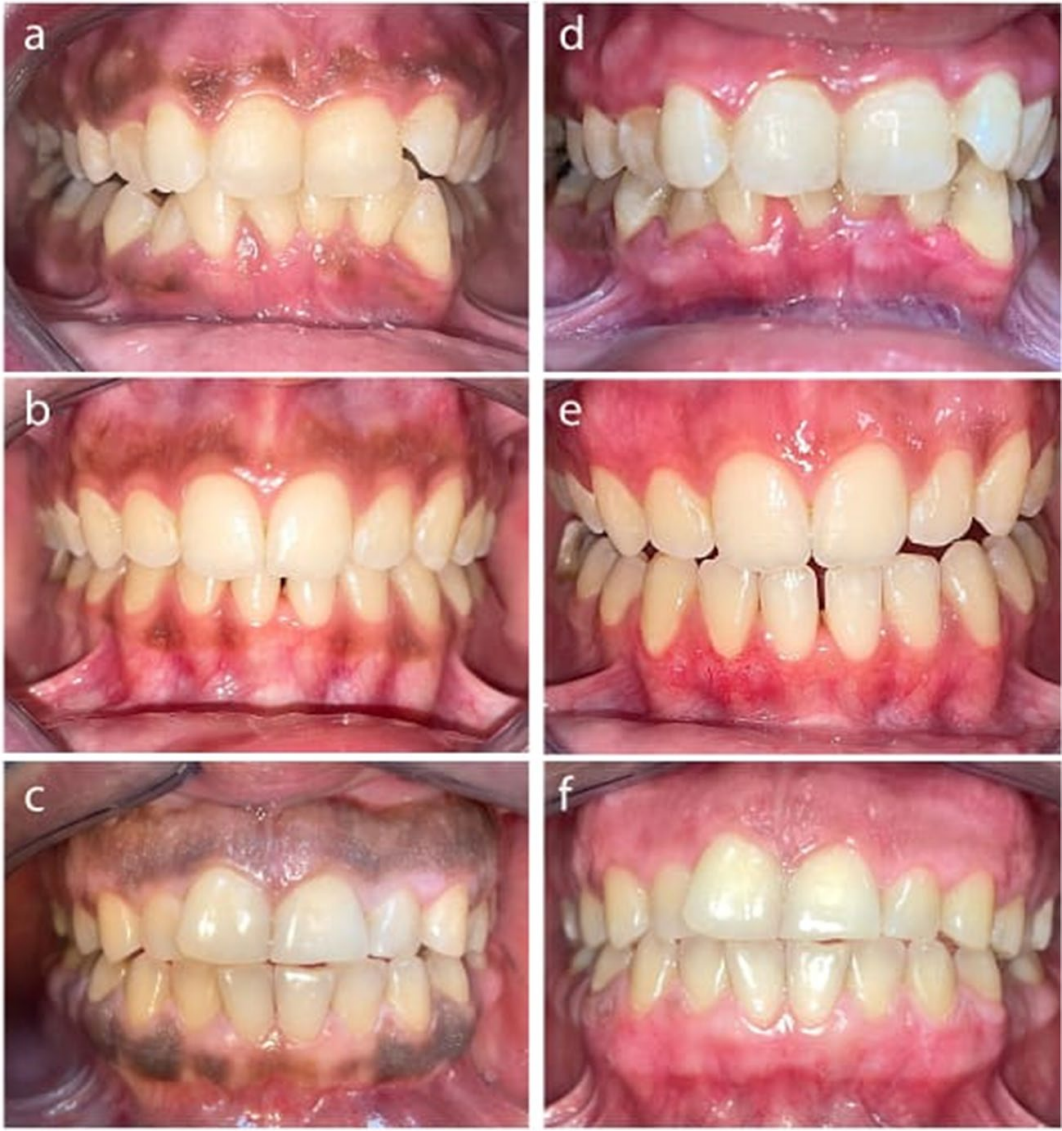
**a** Preoperative view of the surgical scalpel group; **b** preoperative view of the ceramic trimmer bur group; **c** preoperative view of the diode laser group; **d** postoperative view of the surgical scalpel group after 7 days; **e** postoperative view of the ceramic trimmer bur group after 7 days; **f** postoperative view of the diode laser group after 7 days

The use of two indices in the current study was to get more accurate results, as the DOPI is based on color intensity, and the Takashi Index is based on location and extent. An independent observer compared the images taken on the seventh postoperative day with the baseline photo to evaluate them using oral pigmentation indices (Fig. 3).

### Statistical analysis

Data management and statistical analysis were done using the Statistical Package for Social Sciences (IBM® SPSS® Statistics Version 25). Numerical data were described using mean, standard deviation, median, and range. Data were explored for normality by checking the data distribution and using the Kolmogorov–Smirnov and Shapiro–Wilk tests.

Comparisons between groups concerning normally distributed numeric variables (duration of surgery) were performed using the ANOVA test. All other numerical variables were non-parametric and were compared using the Kruskal–Wallis test. A comparison between different observations for the same group before and after treatment was carried out employing the Wilcoxon test.

Qualitative data were presented as frequencies and percentages and analyzed using chi-square and Fisher’s exact tests. All *p* values are two-sided. *p* values < 0.05 were considered significant.

## Results

Twenty-four participants with gingival hyperpigmentation completed the study after 1 week of observation. None of the individuals left or stopped receiving treatment during the follow-up intervals. No patients reported unbearable pain, significant swelling, or adverse symptoms that warranted exclusion. Without excluding anyone, the participants’ collected data were statistically analyzed.

The comparison between groups at each time point (12 h, 4 days, and 7 days) and across the various time points for each group was performed on the variables. There was no statistically significant difference at baseline between groups for all clinical parameters.

### Pain-VAS score

About the median scores for pain, all the tested groups significantly declined over time until they all approached 0 at the 7th-day follow-up (*p* = 0.001). The lowest median pain score was recorded by the laser group at the 12-h follow-up (1.5), followed by the ceramic trimmer bur group (2.5), and finally, the scalpel group (3.5). The laser group revealed a significant decrease in the median pain score compared to the surgical scalpel group (*p* = 0.003) but without significance compared to the ceramic bur group. The median pain score between the groups did not differ significantly at the 4th- or 7th-day follow-ups (*p* = 0.7 and *p* = 1, respectively).

### Gingival bleeding during operation

The scalpel group recorded a significantly (*p* = 0.001) higher median value (2) for the bleeding index. In contrast, the ceramic trimmer bur group recorded a median value (0), and the laser group recorded a median value (0). The ceramic bur and diode laser groups recorded a statistically significant difference compared to the surgical scalpel group.

### Duration of operation

Concerning the time taken to perform the depigmentation procedure, the scalpel, the ceramic trimmer bur, and the laser groups had mean times of 20 ± 1.95, 21.75 ± 1.58, and 11.88 ± 1.34, respectively. The laser group had a significant drop in the time to finish the treatment procedure, different from the scalpel and ceramic trimmer bur groups (*p* = 0.004 and *p* 0.001, respectively).

### The degree of epithelialization

Initially, all groups had partial epithelialization on the fourth day. However, on the seventh day, the percentage of cases that demonstrated complete epithelialization was 87.5% for the scalpel group versus 75% for the other groups, with no statistically significant difference being recorded between any of the groups.

### Wound healing

The median healing value was (5) for the scalpel and ceramic trimmer bur groups and (4) for the laser, with no statistically significant difference (*p* = 0.5) between the groups.

### Changes in intensity of pigmentation (DOPI)

The clinical scoring of gingival pigmentation revealed that DOPI decreased significantly 1 week after treatment in the upper arch (*p* values for the scalpel, ceramic trimmer bur, and laser groups were 0.007, 0.01, and 0.01, respectively) and in the lower arch (*p* values for the scalpel, ceramic trimmer bur, and laser groups were 0.005, 0.01, and 0.007, respectively). The DOPI scores across the three groups at baseline, at 1-week follow-ups, and the percentage of change did not differ significantly.

### Changes in the extension of pigmentation (Takashi Index)

The clinical scoring of gingival pigmentation revealed that Takashi Index scores decreased significantly 1 week after treatment in the upper arch (*p* values for the scalpel, ceramic trimmer bur, and laser groups were 0.007, 0.007, and 0.009, respectively) and in the lower arch (*p* values for the scalpel, ceramic trimmer bur, and laser groups were 0.009, 0.01, and 0.008, respectively). The Takashi Index scores across the three groups at baseline, at 1-week follow-ups, and the percentage of change did not differ significantly.

## Discussion

Black gum complaints and requests for depigmentation are popular, even though none of these symptoms poses a medically problematic risk. Many treatment modalities are available for gingival depigmentation, including the scalpel technique, cauterization, cryotherapy, diamond burs, and lasers [26–29].

Using scalpel for depigmentation offers certain advantages, such as faster healing, lower cost, and the need for fewer instruments. However, scalpel surgery can cause bleeding during and after the procedure, and it requires the use of a periodontal dressing to protect the area of surgery for 7 to 10 days [30].

The ceramic trimmer mentioned in the literature for gingivoplasty has recently been utilized for depigmentation treatment. Soft tissue trimmers offer advantages such as cost-effectiveness, easy availability, and patient acceptance for procedures like depigmentation [15].

Laser ablation has been recognized as one of the most effective, comfortable, and dependable techniques for gingival depigmentation [29]. However, the main drawback of laser treatment is the equipment cost compared to more affordable treatments [31].

Lasers can be utilized in both contact and non-contact applications. When operating in contact mode, operators can use Nd:YAG and diode lasers, which provide tactile sensations. These lasers should only be applied with delicate and light strokes to prevent any harm to the deeper layers, and it is important to avoid maintaining them at one particular point for too long. Conversely, the Erbium family, CO2, and contemporary super-pulsed diode lasers can all be operated non-contact. While this method necessitates a high level of precision from the operator, it provides a more comfortable experience for patients throughout the surgery by removing the scraping sensation, as explained earlier by Muruppel et al. (2020) [13].

The effectiveness of CO2 and diode lasers was compared in a previous study by Moeintaghavi et al. (2022). The diode laser was considered much more aesthetically appealing at the 6-month follow-up [32]. In addition, when the diode and Er:YAG lasers’ effectiveness in gingival depigmentation was compared by Jnaid Harb et al. (2021), no significant aesthetic differences were observed between the two lasers’ outcomes. However, the diode significantly attenuated pain and enhanced hemostasis [33]. Similar clinical outcomes were obtained by Altayeb et al. (2021) in a different investigation after using Er,Cr:YSGG and diode lasers [1]. According to previous studies, the utilization of diode laser for depigmentation is a tolerable and effective treatment that reduces patient anxiety and produces better results [34–36]. Nevertheless, specific care should be taken to prevent the risk of gingival fenestration or bone exposure. The CO2, Er:YAG, and Er, Cr:YSGG lasers have been utilized in previous studies in this field; however, the diode laser provides certain benefits, including increased efficacy, shorter treatment times, and improved coagulation [18, 37–39].

There is not enough research or evidence on the effectiveness of soft tissue trimmers compared to lasers and traditional surgery in reducing discomfort postsurgery and accelerating the wound healing process. Therefore, the present study compared the efficacy of a scalpel, ceramic trimmer bur, and diode laser (980 nm) for gingival depigmentation.

In the current study, a diode laser of 980 nm was used to accomplish laser-assisted gingival depigmentation. Since the diode laser’s approximate wavelength extends from 800 to 1000 nm, it exhibits the highest absorption rate for melanin and the lowest absorption rate for hydroxyapatite in this spectrum [40]. The diode laser is an effective surgical soft tissue laser suited to cutting and coagulating gingival and oral mucosa since it does not impact dental hard tissues. The diode laser’s “hot tip” effect, brought on by heat buildup at the fiber tip, forms a deep coagulation layer in the treated area [41].

The treatment was conducted in contact mode, allowing for good tactile sensation and operating precision [42, 43]. Clinical outcomes demonstrated practical, efficient, and safe ablation of melanin pigmentation using this contact technique. Additionally, achieving complete de-epithelialization necessitates employing the device in contact mode to ensure optimal control of the laser beam without causing harm to nearby teeth and alveolar bone [44].

The Visual Analog Scale was employed in the current study to measure postoperative pain. The present study revealed a statistically significant decrease in the three groups’ median pain scores over time. The laser group reported the least pain at 12-h follow-up, followed by the ceramic bur and scalpel groups. The median pain score was significantly lower in the diode laser group than in the surgical scalpel group but not significantly different from the ceramic bur group. On days 4 and 7, there was no statistically significant difference regarding how each group perceived pain (Table 1).

**Table 1:** The statistical intragroup (Wilcoxon-signed rank test) and intergroup (Kruskal–Wallis test) comparison of pain score (VAS) between surgical scalpel, ceramic trimmer bur, and diode laser groups at the 12-h, 4-day, and 7-day follow-ups

Time	Technique			
	Surgical scalpel	Ceramic trimmer bur	Diode laser	*p* value
12 h: median (mini–max)	3.5 (2–5)	2.5 (2–4)	1.5 (1–3)^a^	0.003*
4 days: median (mini–max)	0 (0–1)	0 (0–1)	0 (0–1)	0.7 ns
7 days: median (mini–max)	0 (0–0)	0 (0–0)	0 (0–0)	1 ns
*p* value	0.001*	0.001*	0.001*	

*Significant *p* < 0.05; *ns*, non-significant

^a^Denotes a statistically significant difference compared to the surgical scalpel group

It was theorized that the more invasive surgical procedures using the scalpel, which result in significant bleeding and surgical incisions that are fully exposed, might be to blame for the higher pain perception associated with them. Since the open wound heals with secondary intention, adding to the postoperative discomfort. Additionally, the increased pain on the tissue surface could be attributed to exposed nerve endings caused by making a deeper incision with a surgical scalpel and the longer procedure time. The coagulation of protein on the wound surface, acting as a biological wound dressing, is why the laser groups experience reduced postoperative pain. Additionally, the laser can seal sensory nerve endings [37, 45]. When using the ceramic trimmer to remove tissue, the bur would need to rotate at a high speed. Through mechanical force and friction, the bur cuts or abrades the tissue when it comes into contact with it. The effectiveness and depth of tissue removal depend on the rotational speed and pressure. It was assumed that the less painful surgical method was using a ceramic trimmer bur maybe because it just removes the epithelium surface’s outermost layer, sparing the lamina propria beneath it, which contains the nerves [46].

The results of the current study are in agreement with the one by Negi et al. (2019), which compared the effectiveness of a soft tissue trimmer and laser for gingival depigmentation and revealed that most laser-treated sites showed minimal to no pain, while patients who underwent bur treatment experienced minimal to moderate pain [15]. In contrast to the results of our study, Goldar et al. (2020) discovered that, although there were no statistically significant differences between the laser and ceramic trimmer groups, subjects who used the laser experienced more discomfort on the first and third postoperative days [10]**.**

Moreover, the outcomes of the current inquiry are in accord with those of Suragimah et al. (2016), who discovered that even though there was no variation between the scalpel and laser techniques on the seventh day, patients who underwent laser treatment initially endured less postoperative pain than those who underwent the scalpel procedure [47].

In the current study, surgical bleeding was compared across all techniques. In contrast to the ceramic bur group and the laser group, the scalpel group’s bleeding median value was statistically significantly more remarkable, without a statistically significant difference between the ceramic bur group and the laser group (Table 2).

**Table 2:** The statistical intergroup comparison of bleeding index (Kruskal–Wallis test) between surgical scalpel, ceramic trimmer bur, and diode laser groups during surgery

Bleeding	Technique			
	Surgical scalpel	Ceramic trimmer bur	Diode laser	*p* value
Bleeding index during surgery Median (mini–max)	2 (1–2)	0 (0–2)^a^	0 (0–1)^a^	0.001*

*Significant *p* < 0.05; *ns*, non-significant

^a^Denotes a statistically significant difference compared to the surgical scalpel group

The “hot tip” effect brought on by heat buildup at the fiber’s end helps remove the superficial layer of epithelium without bleeding or trauma to the mucosa. Furthermore, the blood vessels surrounding tissue up to a diameter of 0.5 mm were found to be sealed by laser. As a result, a thick coagulation layer forms on the treated surface, causing hemostasis and a reasonably dry surgical area [10]. Similarly, these trimmers comprise mixed ceramics, including zircon dioxide that partially stabilized Yttrium and aluminum ceramic. They secure a fine, delicate incision while the heat buildup produces good hemostasis, resulting in minimal bleeding and almost no risk of necrosis. This may be due to the thermo-coagulation impact of the bur-induced heat [15]. The study’s findings corroborated those of Suragimah et al. (2016), who reported that areas treated with lasers bled significantly less than areas treated with scalpels [47]. These findings are consistent with those of an earlier investigation by Al Mokadem et al. (2023), who found that using a soft tissue trimmer during gingivectomy and gingivoplasty surgeries could be a promising and quick method with minimal postoperative pain. They also found that immediate coagulation while using the soft tissue trimmer reduces intraoperative bleeding and enhances wound healing [48].

Regarding the time taken to complete the procedure, it was found that the laser technique consumed significantly less time than the scalpel and ceramic bur techniques. However, a statistically significant difference was not seen when comparing the ceramic bur and scalpel techniques (Table 3). This might be explained by the lack of bleeding throughout the laser surgical procedure, which was simpler and less technique-sensitive than a scalpel. In contrast, ceramic trimmer burs mechanically cut the pigmented tissues, requiring repeated treatment passes to remove the superficial layers of pigmented tissues with the burs, adding time to the process.

**Table 3:** The statistical intergroup comparison of surgery duration (min) (ANOVA test) between surgical scalpel, ceramic trimmer bur, and diode laser groups

Time	Technique			
	Surgical scalpel	Ceramic trimmer bur	Diode laser	*p* value
Duration Mean ± SD Median (mini–max)	20 ± 1.95 21 (11–26)	21.75 ± 1.58 20 (17–30)	11.88 ± 1.34^a,b^ 10.5 (8–18)	< 0.001*

*Significant *p* < 0.05; *ns*, non-significant

^a^Denotes a statistically significant difference compared to the surgical scalpel group

^b^Denotes a statistically significant difference compared to the ceramic bur group

The hydrogen peroxide test was used to assess the degree of epithelialization. In the connective tissue bed underneath the epithelium, catalase reacts with H_2_O_2_ to release H_2_O and O_2_, which are detected as bubbling at the healing site [49]. Bubble formation is not clinically apparent when the epithelial barrier is intact, as H_2_O_2_ cannot reach the connective tissue bed.

The results of the present study showed that on day 4, all groups displayed partial epithelialization; however, on day 7, the scalpel group showed complete epithelialization in 87.5% of the cases, compared to 75% of the cases in all other groups, with no significant difference between any of the groups (*p* = 0.9) (Table 4).

**Table 4:** The statistical intragroup and intergroup comparisons of the degree of epithelialization (chi-square test) between surgical scalpel, ceramic trimmer bur, and diode laser groups at 4-day and 7-day follow-ups

			Surgical scalpel	Ceramic trimmer bur	Diode laser	*p* value (between groups)
Epithelialization, 4 days	Complete	Count % within groups	0 0%	0 0%	0 0%	1 ns
	Partial	Count % within groups	8 100.00%	8 100.00%	8 100.00%	
Epithelialization, 7 days	Complete	Count % within groups	7 87.5%	6 75%	6 75%	0.9 ns
	Partial	Count % within groups	1 12.5%	2 25%	2 25%	

*ns*, non-significant

After depigmentation, the reported time for complete epithelialization varies. In contrast to our findings, Nagpal et al. (2015) found that it took 3 months for gingival depigmentation using a diode laser with 810-nm wavelength to epithelialize completely [50]. A second study was conducted by Jagannathan et al. (2020) utilizing a diode laser with an 810-nm wavelength, and complete epithelialization was also reported 2 weeks after the end of the treatment [17]. The inconsistency in the results between the studies could be attributed to using different laser parameters.

Unlike other research in this area [27, 51, 52], the scalpel and ceramic bur groups had higher median values for gingival healing than the laser group. However, the difference between the groups’ median values did not approach a statistically significant level, and gingival healing was almost complete after 1 week (Table 5). Sharon et al. (2000) and Esen et al. (2004) concluded a 2-week and 6-week duration for postoperative healing following depigmentation with CO2 and cryosurgery, respectively. At the same time, Atsawasuwan et al. (2000) claimed that with the administration of a Nd:YAG laser, full healing was achieved in 1–3 weeks.

**Table 5:** The statistical intergroup comparison of the healing index (Kruskal–Wallis test) between surgical scalpel, ceramic bur, and diode laser groups at 7-day follow-up

Healing	Technique			
	Surgical scalpel	Ceramic trimmer bur	Diode laser	*p* value
Healing index mean ± SD, Median (mini–max)	4.75 ± 0.46 5 (4–5)	4.63 ± 0.52 5 (4–5)	4.37 ± 0.51 4 (4–5)	0.5 ns

*ns*, non-significant

The current study’s findings are consistent with a work by Govila et al. (2011) that employed a 940-nm diode laser for gingival depigmentation [53]. A previous study by Suragimah et al. (2016) that found no statistically significant difference in wound healing between scalpel and laser treatments after gingival depigmentation confirms the results of the current investigation [47]. It has been proposed that this impact is caused by reactive oxygen species (ROS) generation when tissues are exposed to laser light [54]. Additionally, photobiomodulation (PBM) might accelerate the healing of laser-treated wounds. Combining LLLT with high-level laser therapy at the target tissue’s boundary explains lasers’ benefits in this application.

In general, evaluation on day 7 for all procedures showed restoration of the gingiva’s normal features without developing scars. Therefore, regardless of the methods employed, the depigmented gingiva healed uneventfully, irrespective of the techniques used.

Images for the current study were taken using the same camera, zoom, and preset chair position to establish uniformity. The distance between the patient and the camera was likewise standardized by putting the camera on top of a tripod.

The effectiveness of the depigmentation treatment was assessed at baseline and after 1 week using DOPI and Takashi indices to give more accurate results. There was no statistically significant difference between any of the other groups before, after, or in the percentage of change (percentage decrease). However, both melanin scores (DOPI and Takashi) within the same group decreased statistically significantly 1 week after treatment (Tables 6 and 7). The outcomes revealed the absence of a statistically significant difference in gingival depigmentation efficacy between any of the groups. Research by Kumar et al. (2013) and Abdullah et al. (2014) support these findings [55, 56].

**Table 6:** The statistical intragroup (Wilcoxon-signed rank test) and intergroup (Kruskal–Wallis test) comparisons of the Dummett-Gupta Oral Pigmentation Index (DOPI) between surgical scalpel, ceramic trimmer bur, and diode laser groups at baseline and 7-day follow-ups

DOPI		Surgical scalpel	Ceramic trimmer bur	Diode laser	*p* value (between groups)
Upper	Before	2 (1–2)	2 (1–3)	2 (1–3)	0.9 ns
	After 1 week	0 (0–0)	0 (0–1)	0 (0–1)	0.3 ns
*p* value		0.007*	0.01*	0.01*	
Percent change		100 ± 0	93.75 ± 17.67	93.75 ± 17.67	0.7 ns
Lower	Before	2 (2–2)	2 (1–3)	2 (2–3)	0.9 ns
	After 1 week	0 (0–0)	0 (0–1)	0 (0–0)	0.3 ns
*p* value		0.005*	0.01*	0.007*	
Percent change		100 ± 0	93.75 ± 17.67	100 ± 0	0.4 ns

*DOPI*, Dummett-Gupta Oral Pigmentation Index

*Significant *p* < 0.05; *ns*, non-significant

**Table 7:** The statistical intragroup (Wilcoxon-signed rank test) and intergroup (Kruskal–Wallis test) comparisons of the Takashi Index between surgical scalpel, ceramic trimmer bur, and diode laser groups at baseline and 7-day follow-ups

Takashi		Surgical scalpel	Ceramic trimmer bur	Diode laser	*p* value
Upper	Before	2 (1–2)	2 (2–2)	2 (1–2)	0.3 ns
	After 1 week	0 (0–0)	0 (0–1)	0 (0–1)	0.3 ns
*p* value		0.007*	0.007*	0.009*	
Percent change		100 ± 0	93.75 ± 17.67	93.75 ± 17.67	0.8 ns
Lower	Before	2 (1–2)	2 (2–2)	2 (1–2)	0.06 ns
	After 1 week	0 (0–0)	0 (0–1)	0 (0–0)	0.3 ns
*p* value		0.009*	0.01*	0.008*	
Percent change		100 ± 0	93.75 ± 17.67	100 ± 0	0.4 ns

*Significant *p* < 0.05; *ns*, non-significant

However, given the short follow-up period of this study and because the outcomes of depigmentation procedures are dependent not only on the level of achieved depigmentation but also on how long it takes for re-pigmentation to develop, these results may not be conclusive, and more studies with a prolonged follow-up duration are recommended.

## Conclusion

The surgical scalpel, ceramic trimmer bur, and diode laser 980 nm efficiently removed the gingival pigments, and the clinical outcomes were comparable 1-week postsurgery. A diode laser is a safe and efficient treatment option for gingival hyperpigmentation. However, in comparison to a diode laser, considering that using a soft tissue trimmer is simple and readily available and costs just one-sixtieth of what a laser does, it can be said that it can be utilized as an alternative to a laser in gingival depigmentation procedures.

## Data Availability

The data that support the findings of this study are available on request from the corresponding author.

## Footnotes

1 Ubistesin™ 1/200,000, 3 M ESPE AG, Germany.

2 DFS Diamon Precicut® tissue trimmer, Germany.

3 Doctor Smile Diode Laser, LAMPDA, Italy.

## References

[1] Altayeb W, Hamadah O, Alhaffar BA, Abdullah A, Romanos G (2021). Gingival depigmentation with diode and Er, Cr: YSGG laser: evaluating re-pigmentation rate and patient perceptions. Clin Oral Invest.

[2] Bakhshi M, Rahmani S, Rahmani A (2015). Lasers in esthetic treatment of gingival melanin hyperpigmentation: a review article. Lasers Med Sci.

[3] Tsatmali M, Ancans J, Thody AJ (2002). Melanocyte function and its control by melanocortin peptides. J Histochem Cytochem.

[4] Grover HS, Dadlani H, Bhardwaj A, Yadav A, Lal S (2014). Evaluation of patient response and recurrence of pigmentation following gingival depigmentation using laser and scalpel technique: a clinical study. Journal of Indian Society of Periodontology.

[5] Jha N, Ryu JJ, Wahab R, Al-Khedhairy AA, Choi EH, Kaushik NK (2017). Treatment of oral hyperpigmentation and gummy smile using lasers and role of plasma as a novel treatment technique in dentistry An introductory review. Oncotarget.

[6] Roshna T, Nandakumar K (2005). Anterior esthetic gingival depigmentation and crown lengthening: report of a case. J Contemp Dent Pract.

[7] Gupta G, Kumar A, Khatri M, Puri K, Jain D, Bansal M (2014). Comparison of two different depigmentation techniques for treatment of hyperpigmented gingiva. J Ind Soc Periodontol.

[8] Amaral M, De Ávila J, Abreu M, Mesquita R (2015). Diode laser surgery versus scalpel surgery in the treatment of fibrous hyperplasia a randomized clinical trial. Int J Oral Maxillofacial Surg.

[9] Haytac MC, Ozcelik O (2006). Evaluation of patient perceptions after frenectomy operations a comparison of carbon dioxide laser and scalpel techniques. J Periodontol.

[10] Goldar K, Chaubey KK, Agarwal S, Agarwal T (2020). Gingival depigmentation by gingival ceramic trimmer. University J Dental Sci.

[11] Parker S (2007). Laser regulation and safety in general dental practice. Br Dent J.

[12] Valenti C, Pagano S, Bozza S, Ciurnella E, Lomurno G, Capobianco B, Coniglio M, Cianetti S, Marinucci L (2021). Use of the Er: YAG laser in conservative dentistry: evaluation of the microbial population in carious lesions. Materials.

[13] Muruppel AM, Pai BSJ, Bhat S, Parker S, Lynch E (2020). Laser-assisted depigmentation-an introspection of the science, techniques, and perceptions. Dent J (Basel).

[14] Giannelli M, Formigli L, Bani D (2014). Comparative evaluation of photoablative efficacy of erbium: yttrium-aluminium-garnet and diode laser for the treatment of gingival hyperpigmentation A randomized split-mouth clinical trial. J Periodontol.

[15] Negi R, Gupta R, Dahiya P, Kumar M, Bansal V, Samlok JK (2019). Ceramic soft tissue trimming bur: a new tool for gingival depigmentation. J Oral Biol Craniofacial Res.

[16] Chhina S, Gakhar A, Gupta S, Ss S, Sharma E, Arora SA (2019). Assessment of clinical outcomes and patient response to gingival depigmentation by scalpel surgical stripping and diode laser: a randomized split-mouth study. J Adv Oral Res.

[17] Jagannathan R, Rajendran S, Balaji TM, Varadarajan S, Sridhar LP (2020). Comparative evaluation of gingival depigmentation by scalpel, electrosurgery, and laser: a 14 months’ follow-up study. J Contemp Dent Pract.

[18] Bakhshi M, Mojahedi SM, Asnaashari M, Rahmani S, Namdari M (2018). Gingival depigmentation by Er, Cr: YSGG laser and diode laser: a split mouth, clinical trial study lasers in gingival depigmentation. Laser therapy.

[19] Varghese KG, Manoharan S, Sadhanandan M (2015). Evaluation of bleeding following dental extraction in patients on long-term antiplatelet therapy: a clinical trial. Indian J Dent Res.

[20] Marucha PT, Kiecolt-Glaser JK, Favagehi M (1998). Mucosal wound healing is impaired by examination stress. Psychosom Med.

[21] Bansal M, Kumar A, Puri K, Khatri M, Gupta G, Vij H (2016). Clinical and histologic evaluation of platelet-rich fibrin accelerated epithelization of gingival wound. J Cutan Aesthet Surg.

[22] Chawla K, Lamba AK, Tandon S, Faraz F, Gaba V (2016). Effect of low-level laser therapy on wound healing after depigmentation procedure: a clinical study. J Ind Soc Periodontol.

[23] Pippi R (2017). Post-surgical clinical monitoring of soft tissue wound healing in periodontal and implant surgery. Int J Med Sci.

[24] Dummett CO, Gupta OP (1964). Estimating the epidemiology of oral pigmentation. J Natl Med Assoc.

[25] Hanioka T, Tanaka K, Ojima M, Yuuki K (2005). Association of melanin pigmentation in the gingiva of children with parents who smoke. Pediatrics.

[26] Murthy MB, Kaur J, Das R (2012). Treatment of gingival hyperpigmentation with rotary abrasive, scalpel, and laser techniques: a case series. Journal of Indian Society of Periodontology.

[27] Atsawasuwan P, Greethong K, Nimmanon V (2000). Treatment of gingival hyperpigmentation for esthetic purposes by Nd: YAG laser: report of 4 cases. J Periodontol.

[28] Shah C, Dave R, Shah M, Dave D (2014). Evaluation of scalpel versus diode laser for gingival depigmentation: a case report. Int J Adv Health Sci.

[29] Berk G, Atici K, Berk N (2005). Treatment of gingival pigmentation with Er, Cr: YSGG laser. J Oral Laser Appl.

[30] Javali MA, Roopali T, Deshmukh J (2011). Esthetic management of gingival hyperpigmentation: report of two cases. Int J Dent Clin.

[31] El Shenawy HM, Nasry SA, Zaky AA, Quriba MA (2015). Treatment of gingival hyperpigmentation by diode laser for esthetical purposes. Open access Macedonian J Med Sci.

[32] Moeintaghavi A, Ahrari F, Fallahrastegar A, Salehnia A (2022). Comparison of the effectiveness of CO2 and diode lasers for gingival melanin depigmentation: a randomized clinical trial. J Lasers Med Sci.

[33] Jnaid Harb ZK, El-Sayed W, Alkhabuli J (2021) Gingival depigmentation using diode 980 nm and erbium-YAG 2940 nm lasers: A split-mouth clinical comparative study. International Journal of Dentistry. 2021:9424793. 10.1155/2021/9424793 10.1155/2021/9424793 PMC8727139 34992657

[34] Chagra J, Bouguezzi A, Sioud S, Hentati H, Selmi J (2020). Gingival melanin depigmentation by 808 nm diode laser: report of a case. Case Reports in Dentistry.

[35] Jokar L, Bayani M, Hamidi H, Keivan M, Azari-Marhabi S (2019). A comparison of 940 nm diode laser and cryosurgery with liquid nitrogen in the treatment of gingival physiologic hyperpigmentation using split mouth technique: 12 months follow up. Journal of Lasers in Medical Sciences.

[36] Mojahedi SM, Bakhshi M, Babaei S, Mehdipour A, Asayesh H (2018). Effect of 810 nm diode laser on physiologic gingival pigmentation. Laser therapy.

[37] Kaya GŞ, Yavuz GY, Sümbüllü MA, Dayı E (2012). A comparison of diode laser and Er: YAG lasers in the treatment of gingival melanin pigmentation. Oral Surg Oral Med Oral Pathol Oral Radiol.

[38] Rastegar S, Jacques SL, Motamedi M, Kim B-M (1992) Theoretical analysis of equivalency of high-power diode laser (810 nm) and Nd: YAG laser (1064 nm) for coagulation of tissue. Proc SPIE 1646:150–160

[39] Esmaeili S, Shahbazi S, Asnaashari M (2022). Gingival melanin depigmentation using a diode 808-nm laser: a case series. Journal of Lasers in Medical Sciences.

[40] Al Timimi ZJ, Alhabeel MS (2019) Laser dental treatment techniques. In: Sivapatham S (ed) Prevention, detection and management of oral cancer. IntechOpen. 10.5772/intechopen.80029

[41] Ozbayrak S, Dumlu A, Ercalik-Yalcinkaya S (2000). Treatment of melanin-pigmented gingiva and oral mucosa by CO2 laser. Oral Surg, Oral Med, Oral Pathol, Oral Radiol, Endodontol.

[42] Rosa DSA, Aranha ACC, de Paula EC, Aoki A (2007). Esthetic treatment of gingival melanin hyperpigmentation with Er: YAG laser: short-term clinical observations and patient follow-up. J Periodontol.

[43] Ishii S, Aoki A, Kawashima Y, Watanabe H, Ishikawa I (2002). Application of an Er: YAG laser to remove gingival melanin hyperpigmentation: treatment procedure and clinical evaluation. J Jpn Soc Laser Dent.

[44] Giannelli M, Formigli L, Lasagni M, Bani D (2013). A new thermographic and fluorescent method for tuning photoablative laser removal of the gingival epithelium in patients with chronic periodontitis and hyperpigmentation. Photomed Laser Surg.

[45] Bakutra G, Shankarapillai R, Mathur L, Manohar B (2017). Comparative evaluation of diode laser ablation and surgical stripping technique for gingival depigmentation: a clinical and immunohistochemical study. Int J Health Sci.

[46] Goldar K, Agarwal S, Bharali J, Agarwal T (2021). Gingival ceramic trimmer-a wonder device to eliminate gingival hyperpigmentation (for gingival depigmentation) in comparison to laser. International Journal of Dental Science and Innovative Research.

[47] Suragimath G, Lohana MH, Varma S (2016). A split mouth randomized clinical comparative study to evaluate the efficacy of gingival depigmentation procedure using conventional scalpel technique or diode laser. J lasers Med Sci.

[48] AlMokadem MMS, Abdelrahman AR, Nasr SS, Elkhouly S (2023). Clinical efficacy of soft tissue trimmer versus conventional surgical excision of gingival hyperplasia on postoperative pain: a randomized clinical trial. Advanced Dental J.

[49] Bhusari B, Kasat S (2011). Comparison between scalpel technique and electrosurgery for depigmentation: a case series. J Ind Soc Periodontol.

[50] Nagpal D, Prakash S, Singh G (2015). Gingival depigmentation using different techniques: a follow up study. Arch Dent Med Res.

[51] Sharon E, Azaz B, Ulmansky M (2000). Vaporization of melanin in oral tissues and skin with a carbon dioxide laser: a canine study. J Oral Maxillofac Surg.

[52] Esen E, Haytac MC, Öz İA, Erdoğan Ö, Karsli ED (2004). Gingival melanin pigmentation and its treatment with the CO2 laser. Oral Surg, Oral Med, Oral Pathol, Oral Radiol, Endodontol.

[53] Govila V, Gulati M, Govila S (2011). Diode laser Applications in Periodontics. Indian Journal of Dental Sciences.

[54] Niimi H, Ohsugi Y, Katagiri S, Watanabe K, Hatasa M, Shimohira T, Tsuchiya Y, Maekawa S, Hirota T, Kadokura H (2020). Effects of low-level Er: YAG laser irradiation on proliferation and calcification of primary osteoblast-like cells isolated from rat calvaria. Frontiers Cell Developmental Biol.

[55] Abdullah BA, Al-shmaah ZA (2014). The use of ErCr: YSGG versus diode LASER in gingival melanin depigmentation. Int J Enhanc Res Sci Techn Engg.

[56] Kumar S, Bhat GS, Bhat KM (2013). Comparative evaluation of gingival depigmentation using tetrafluoroethane cryosurgery and gingival abrasion technique: two years follow up. J Clin Diagnostic Research: JCDR.

